# Testosterone positively regulates vagina NO-induced relaxation: an experimental study in rats

**DOI:** 10.1007/s40618-022-01743-4

**Published:** 2022-01-24

**Authors:** I. Cellai, S. Filippi, P. Comeglio, S. Cipriani, E. Maseroli, V. Di Stasi, T. Todisco, S. Marchiani, L. Tamburrino, F. Villanelli, S. Vezzani, C. Corno, M. Fambrini, G. Guarnieri, E. Sarchielli, A. Morelli, G. Rastrelli, M. Maggi, L. Vignozzi

**Affiliations:** 1 Andrology, Women’s Endocrinology and Gender Incongruence Unit, Department of Excellence Experimental and Clinical Biomedical Sciences “Mario Serio”, University of Florence, Viale Pieraccini 6, 50134 Florence, Italy; 2 Interdepartmental Laboratory of Functional and Cellular Pharmacology of Reproduction, Department of Neurosciences, Drug Research and Child Health (NEUROFARBA), University of Florence, Viale Pieraccini 6, 50134 Florence, Italy; 3 Department of Experimental and Clinical Medicine, University of Florence, Largo Brambilla 3, 50134 Florence, Italy; 4 Endocrinology Unit, Department of Excellence Experimental and Clinical Biomedical Sciences “Mario Serio”, University of Florence, Viale Pieraccini 6, 50134 Florence, Italy; 5 Division of Obstetrics and Gynecology, Department of Excellence Experimental and Clinical Biomedical Sciences “Mario Serio”, University of Florence, Viale Morgagni 50, 50134 Florence, Italy; 6 I.N.B.B. (Istituto Nazionale Biostrutture e Biosistemi), Viale delle Medaglie d’Oro 305, 00136 Rome, Italy

**Keywords:** Ovariectomy, Estradiol, Testosterone, Vagina, Smooth muscle, Nitric oxide

## Abstract

**Purpose:**

Female sexual response involves a complex interplay between neurophysiological mechanisms and the nitric oxide (NO)-mediated relaxation of clitoris and vagina. The aim of this study was to evaluate sex steroids regulation of the relaxant pathway in vagina, using a validated animal model.

**Methods:**

Subgroups of OVX Sprague–Dawley rats were treated with 17β-estradiol, testosterone, or testosterone and letrozole, and compared with a group of intact animals. Masson’s trichrome staining was performed for morphological evaluation of the distal vaginal wall, in vitro contractility studies investigated the effect of OVX and in vivo treatments on vaginal smooth muscle activity. RNA from vaginal tissue was analyzed by semi-quantitative RT-PCR.

**Results:**

Immunohistochemical analysis showed that OVX induced epithelial and smooth muscle structural atrophy, testosterone and testo + letrozole increased the muscle bundles content and organization without affecting the epithelium while 17β-estradiol mediated the opposite effects. In vitro contractility studies were performed on noradrenaline pre-contracted vaginal strips from each experimental group. Acetylcholine (0.001–10 µM) stimulation induced a concentration-dependent relaxation, significantly reduced by NO-synthase inhibitor L-NAME and by guanylate cyclase inhibitor ODQ. OVX resulted in a decreased responsiveness to acetylcholine, restored by testosterone, with or without letrozole, but not by 17β-estradiol. OVX sensitivity to the NO-donor SNP was higher than in the control. Vardenafil, a PDE5 inhibitor, enhanced SNP effect in OVX + testosterone as well as in control, as supported by RNA expression analysis.

**Conclusions:**

Our study demonstrates that testosterone improves the NO-mediated smooth muscle vaginal cells relaxation confirming its role in maintaining the integrity of muscular relaxant machinery.

## Introduction

Testosterone (T), one of the most potent androgens, makes a quantitative contribution to the circulating sex steroids pool, reaching a level significantly higher than 17β-estradiol (E2), not only in male but also in female [1].

The biological activity of T in women has been considered enigmatic and left overlooked for a long time. However, over the last decade, the attention on this topic has been intensified and mounting evidence pointed to the crucial role of T in female sexuality. In fact, T levels have been positively associated with sexual function in women, and many randomized placebo-controlled trials (RCTs) have shown that T therapy can be an effective treatment for female sexual dysfunction [2]. In particular, the main therapeutic indication for the T prescription is hypoactive sexual desire disorder (HSDD) in surgically and naturally menopausal women [3–5]. Several recent studies have also investigated the use of intravaginal T in postmenopausal women for the treatment of vulvovaginal atrophy and genitourinary syndrome of menopause (GSM) [2, 6, 7], positing vagina as a new target for T. Noteworthy, we recently demonstrated, for the first time, that the enzymatic machinery able to produce androgens—including T and its potent effector dihydrotestosterone (DHT)—is present in the human vagina, substantiating the construct of intracrinology in this organ [8].

However, although the biological mechanism(s) of action of T in the vagina have yet to be systematically explored, recent studies have revealed a complex and multifaceted anti-inflammatory effect of DHT in human vagina cells [9]. This effect is particularly relevant when considering that, due to its anatomical location, the vagina is the most prone to infections and chronic inflammatory diseases among the female genital organs.

Another peculiar characteristic of the vagina is its substantial modification during sexual activity while encompassing rapid but intense muscular relaxation and contraction. Sexual arousal, which is primarily elicited by sensory stimulation triggering multiple brain areas, finally leads to the reduction of central sympathetic tone and to the release of relaxant neurotransmitters, such as acetylcholine (Ach), which act in combination with nitric oxide, NO. Ach and NO interact with each other to determine a rapid increase of blood flow to the genital corpora cavernosa and a muscular relaxation of the vaginal wall. In particular, NO produced either by non‐adrenergic, non‐cholinergic (NANC) fibers or by the endothelium after cholinergic stimulation—triggers guanylyl cyclase (GCs), which converts GTP to cyclic GMP (cGMP). Then, cGMP-dependent protein kinase (PRKG) stimulation leads to intracellular calcium reduction and smooth muscle relaxation [10–13]. Intracellular cGMP levels are also finely regulated by phosphodiesterase type 5 (PDE5), which is involved in cGMP cleavage and degradation, thus breaking down the smooth muscle relaxation. Several human urogenital districts, including bladder, clitoris and vagina express a high PDE5 activity [14–17]. Interestingly, recent studies demonstrated that T-independently from its conversion into estradiol-positively regulates clitoral NO-dependent relaxant pathway in some experimental models [18]. However, a similar positive effect of T on vaginal relaxation induced by the vasoactive intestinal polypeptide (VIP), has been reported so far [19, 20], but T actions on the other relevant relaxant and contractile pathways have not yet been completely unraveled. In addition, whether this positive effect of T on vaginal relaxation was most likely caused by its conversion to estradiol is still an unanswered question, thus requiring further investigation.

The present study was undertaken to disentangle the differential effect of sex steroids on the muscular relaxant (NO/cGMP/PDE5) pathway in the vagina. Thus, the study was carried out using a previously well-established animal model of ovariectomized female rats, alternatively treated with estradiol, T or T in combination with the aromatase inhibitor, letrozole, the latter to completely block T-conversion into estradiol.

## Materials and methods

Experimental procedures were carried out using the facilities of the Molecular Medicine Facility, Department of Excellence Experimental and Clinical Biomedical Sciences “Mario Serio”, University of Florence, and those of CE.S.A.L. (Centro Stabulazione degli Animali da Laboratorio), Department of Neurosciences, Psychology, Drug Research and Child Health (NEUROFARBA), University of Florence, Italy.

### Animals

Mature female Sprague–Dawley (SD) rats (*n* = 60, 230–250 g, 12 weeks old; Envigo, San Pietro al Natisone, Udine, Italy) were individually caged under standard conditions in a temperature and humidity-controlled room on a 12-h light/dark cycle. Water and food (Global diet; Mucedola srl, Settimo Milanese, Milan, Italy) were unrestricted throughout the study, until sacrifice, performed by beheading.

One week later, the animals were randomly assigned to two different groups: intact (control, *n* = 18) and ovariectomized rats (*n* = 42). Female rats were bilaterally ovariectomized under ketamine and xylazine (75 and 10 mg/kg, respectively, injected intraperitoneally). After anesthesia, the animal was placed in ventral recumbence with the tail towards the surgeon. The dorsal mid-lumbar area was shaved and swabbed with surgical scrub, iodine, and alcohol. A 2–3 cm dorsal midline skin incision was made halfway between the caudal edge of the ribcage and the base of the tail. A single incision (5.5–10 mm long) was made into the muscle wall on both the right and left sides approximately 1/3 of the distance between the spinal cord and the ventral midline. The ovary and the oviduct were exteriorized through the muscle wall. A hemostat was clamped around the uterine vasculature between the oviduct and uterus and each ovary and part of the oviduct was removed with single cuts through the oviducts near the ovary. The hemostat was removed and the remaining tissue was replaced into the peritoneal cavity. The ovary on the other side was removed in a similar manner and the muscle incision was not sutured, this protocol was previously validated for the establishment of ovariectomized animal models [21]. Two weeks after ovariectomy, a group of ovariectomized rats did not receive any treatment (OVX, *n* = 12) whereas the different hormonal treatments were started in the rest of ovariectomized animals. A first subgroup was treated with subcutaneous injections of 17β-estradiol (E210 μg/kg/day; OVX + E, *n* = 10). A second subset was supplemented with intramuscular injections of T (T, 30 mg/kg, weekly: OVX + T, *n* = 10). The doses of E2 and T were chosen in accordance with the previous studies [22–24]. Finally, a third group of ovariectomized rats (OVX + T + L, *n* = 10) was treated with intramuscular injections of T (30 mg/kg, weekly) and letrozole (L, 2.5 mg/kg/day, dissolved in drinking water). 17β-estradiol and letrozole were purchased from Sigma-Aldrich (St. Louis, MO, USA) and Tocris Biosciences (Bristol, UK) respectively, testosterone was supplied by Bayer-Schering Pharma (Berlin, Germany). After six weeks of hormonal treatment, all the animals were sacrificed, and the vagina harvested for subsequent analysis. Animals were permanently monitored (24/7) regarding their wellbeing, following the ARRIVE (Animal Research: Reporting of In Vivo Experiments) guidelines for reporting animal studies [25].

### Masson trichrome staining

Paraffin-embedded distal vaginal segments were prepared from each experimental group and sectioned with a microtome. For each sample, a 5 µm-thick section was stained with Masson’s Trichrome stain (Bio-Optica, Milan, Italy), following the manufacturer’s instructions, for the morphological examination. The images of the stained sections were visualized and captured under a Nikon Microphot-FXA microscope (Nikon, Tokyo, Japan).

### Contractility studies

To investigate the effect of ovariectomy and in vivo treatments on vagina smooth muscle activity, in vitro contractility studies were carried out as previously described [26]. Briefly, rat vagina strips isolated from each experimental group, were vertically mounted in organ chambers under 0.5 g resting tension, optimal for vaginal rat strips. Preparations were immersed in a 10 ml bath containing a physiological salt solution (NaCl 119.0 mM, KCl 4.6 mM, CaCl_2_ 1.5 mM, MgCl_2_ 1.2 mM, NaHCO_3_ 15 mM, NaH_2_PO_4_ 1.2 mM and glucose 5.5 mM), maintained at 37 °C and aerated with 95% O_2_ and 5% CO_2_. Noradrenaline (NA; Sigma-Aldrich)-induced dose-dependent contraction of vagina strips displayed a maximal contraction at 3 µM, which was therefore used as the fixed dose to test pre-contracted strips in the different experimental groups. The degree of a stable contractile response obtained after 5 min was taken as 100%, and the relaxant effect induced by the presence of increasing concentration (1 nM to 10 µM) of acetylcholine (Ach, Sigma-Aldrich) was referred to this value. In separate experiments, the relaxant effect was also evaluated with or without the presence of guanylyl cyclase inhibitor ODQ (1H-[1, 2, 4] oxadiazolo-[4, 3-a] quinoxalin-1-one; 1 µM; Tocris) or nitric oxide synthase inhibitor L-NAME (N omega-Nitro-L-arginine methyl ester hydrochloride; 100 µM; Sigma-Aldrich). Similar experiments were conducted with increasing concentration (1 nM to 100 µM) of NO-donor sodium nitroprusside (SNP, Sigma-Aldrich), with or without co-treatment with PDE5 inhibitor vardenafil (100 nM, Bayer-Schering Pharma), after pre-contraction with 3 μM NA. Changes in isometric tension were recorded on a chart polygraph (Battaglia Rangoni, San Giorgio di Piano, Bologna, Italy). All reagents were dissolved daily in double-distilled water, and further dilutions of all substances were made in Krebs’ solution. For each experimental set, each point represents the mean ± s.e.m. (standard error of the mean) of n samples for each group.

### Real-time reverse transcriptase PCR

The distal vagina tissues were harvested for RNA extraction after tissue removal. Isolation of total RNA from rat vaginal tissues was performed using TRIzol reagent (Life Technologies, Paisley, UK) and RNeasy Mini Kit (Qiagen, Hilden, Germany), both according to the manufacturers’ instructions. cDNA synthesis was carried out using the iScript™ cDNA Synthesis Kit (Bio-Rad Laboratories, Hercules, CA), using 100 ng of mRNA in 20 µl reaction volume, in accordance with the following protocol: 5 min at 25 °C, 30 min at 42 °C and 5 min at 85 °C.

Semi-quantitative real-time reverse transcriptase PCR (qRT-PCR) amplification and detection were carried out using TaqMan™ Gene Expression Assays (Life Technologies) and the CFX96 Two-Color Real-Time PCR Detection System (Bio-Rad Laboratories) with the following thermal cycler conditions: 40 cycles at 95 °C for 15 s and 60 °C for 1 min. Gene expression analysis was performed using pre-developed assays purchased from Life Technologies (in Table 1 we report the assay list and probes ID). The 18S ribosomal RNA subunit was quantified with a predeveloped assay (Hs99999901_s1, Life Technologies) and used as the housekeeping gene for the relative quantitation of the target genes based on the comparative threshold cycle (Ct) 2^−ΔΔCt^ method [27], with some modifications. In detail, we used the untreated group as the calibrator in each analysis, so that the calculations would provide the fold-change of each treated group relative to the untreated.

**Table 1:** List of predeveloped assays and relative IDs

Rat assay	ID
Nos3 (eNos)	Rn02132634_s1
Nos1 (nNos)	Rn00583793_m1
Prkg1	Rn01451055_m1
Gucy1a3	Rn00567252_m1
Gucy1b3	Rn00562775_m1
Pde5a	Rn01639345_m1
Acta2	Rn01759928_g1

*eNos* endothelial nitric oxide synthase; *nNos* neuronal nitric oxide synthase; *Prkg1* cGMP-dependent protein kinase 1; *Gucy1a3* guanylate cyclase soluble subunit alpha-3; *Gucy1b3* guanylate cyclase soluble subunit beta-3; *Pde5a* phosphodiesterase type 5; *Acta2* actin alpha 2, smooth muscle

### Statistical analysis

Results are expressed as mean ± s.e.m. (standard error of the mean). The statistical analysis was performed with a one-way ANOVA test followed by Mann–Whitney *U*-test to evaluate differences between the groups, with *p* < 0.05 considered as significant. Statistical analysis was performed with the Statistical Package for the Social Sciences (version 27.0; SPSS Inc., Chicago, IL) for Windows. Half-maximal response inhibition concentration (IC_50_) values and maximal inhibitory effect (*I*_max_), values were calculated using the computer program ALLFIT [28].

## Results

### Analysis of epithelial and smooth muscle structural organization in histological sections of rat distal vagina

**Fig. 1:**
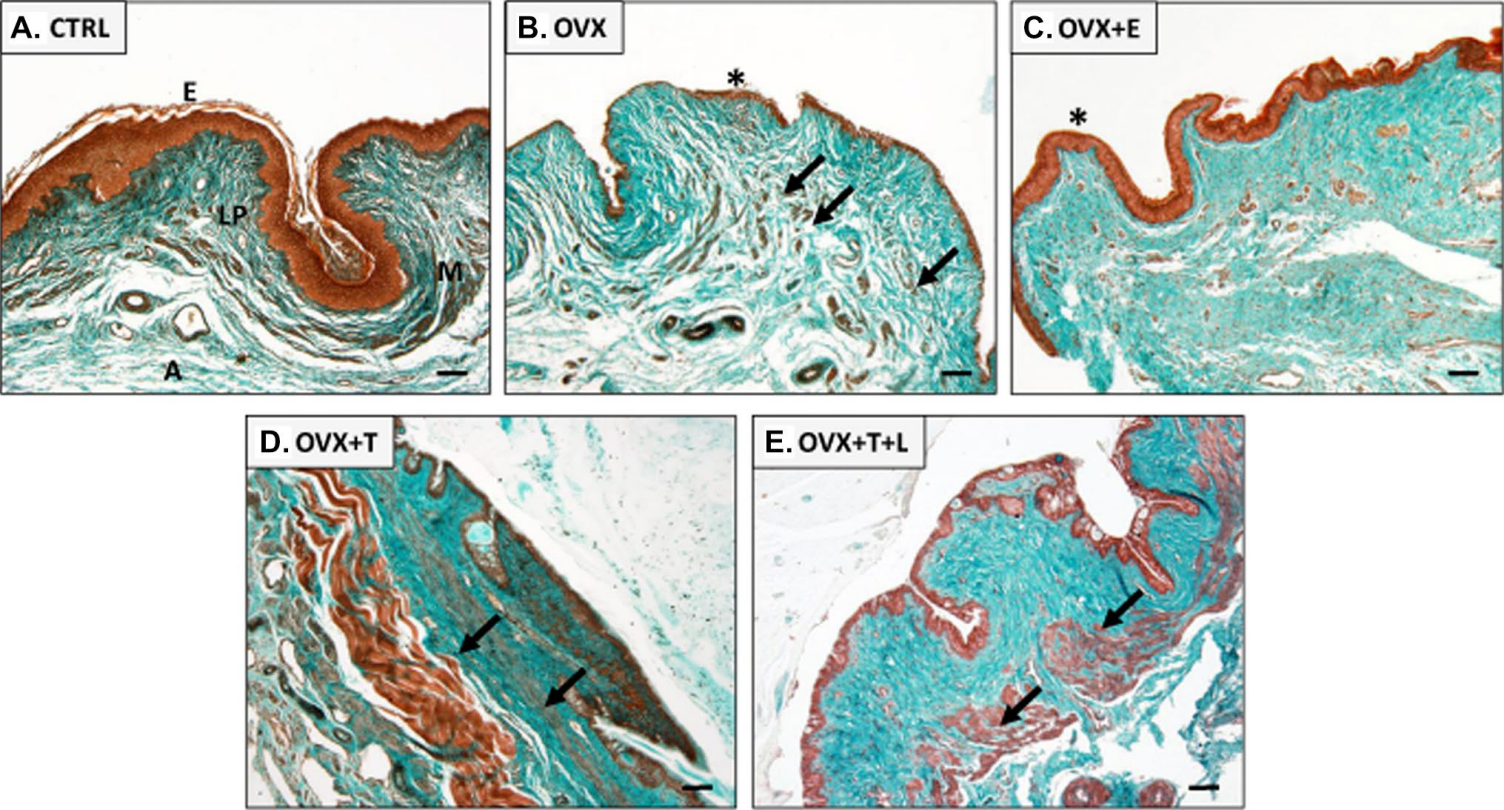
Representative images of Masson’s Trichrome staining of the epithelium (E), lamina propria (LP), muscularis (M) and adventitia (A) at the distal portion of rat vaginal wall. A normal histological structure is detectable for the control group (panel **A**). An evident epithelium atrophy (asterisk) and reduced, poorly organized, smooth muscle bundles (arrows) are visible in OVX section (panel **B**). Epithelium reconstitution (asterisk), with no effects on the muscularis by E2 treatment is shown (panel **C**). T (panel **D**) and T + L (panel **E**) treatments clearly increase the muscularis layer (arrows), without reverting the epithelium atrophy. Scale bar = 100 µm

Masson’s trichrome staining was performed to evaluate the histological structure of the distal portion of the rat vaginal wall, which, similarly to humans, comprises the following four layers: epithelium, collagen-rich lamina propria, muscularis containing smooth muscle bundles, and adventitia, as shown in Fig. 1 (panel A). Compared to control, an evident atrophy of the epithelial layer was detected in the OVX group (Fig. 1, panel B). In addition, no changes were observed in the lamina propria, while the smooth muscle bundles in the muscularis appeared smaller and poorly organized after ovariectomy (Fig. 1, panel B). E2 treatment was able to restore the epithelium thickness, without changing the muscularis content and organization (Fig. 1, panel C), as compared to OVX section. Conversely, both T and T + L treatments determined a positive effect on smooth muscle component that appeared increased and better organized, while no effect was evident on the epithelium, as compared to OVX (Fig. 1, panels D and E).

### Distal vagina mRNA expression of genes related to relaxant signaling

**Fig. 2:**
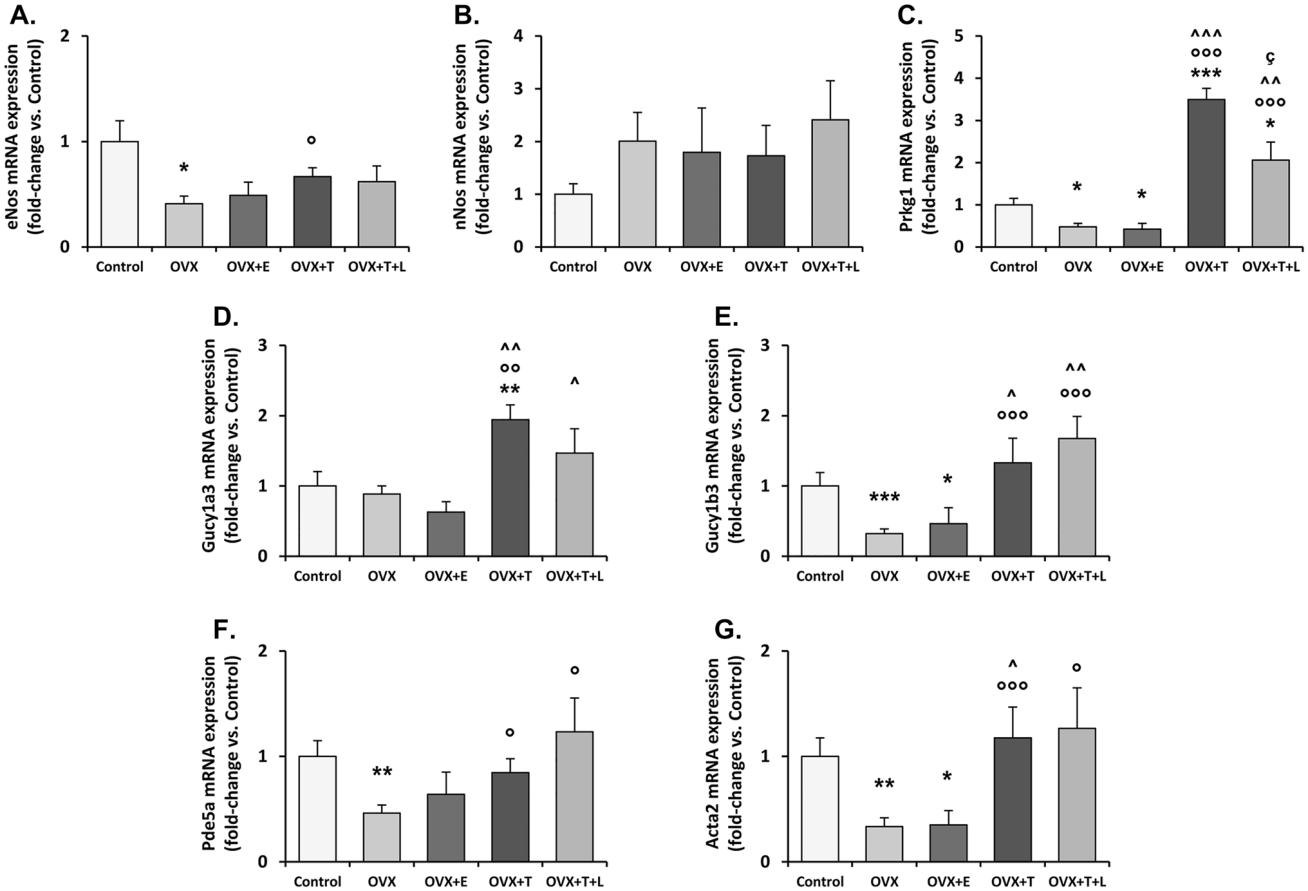
Panels **A**-to-**G**. mRNA expression of NO-signaling-related (eNos, nNos, Prkg1, Gucy1a3, Gucy1b3, Pde5a) and smooth muscle (Acta2) genes in rat female vagina as evaluated by qRT-PCR. Data were calculated according to the 2^−ΔΔCt^ comparative method, with the 18S ribosomal RNA subunit used as housekeeping gene for normalization and are reported in fold changes vs. Control group as mean ± s.e.m. (Control, *n* = 18; OVX, *n* = 12; OVX + E, *n* = 6; OVX + T, *n* = 10; OVX + T + L, *n* = 6). Statistical analysis was performed using Mann–Whitney tests, with *p* < 0.05 considered as significant. *p < 0.05, **p < 0.01, ***p < 0.001 vs. Control; °*p* < 0.05, °°*p* < 0.01, °°°*p* < 0.001 vs. OVX. ^*p* < 0.05, ^^*p* < 0.01, ^^^*p* < 0.001 vs. OVX + E; ç *p* < 0.05 vs. OVX + T

Figure 2 shows the effect of sex steroid manipulation on vagina expression of genes related to relaxant signaling in the different experimental groups. OVX downregulated all the genes involved in NO/cGMP/PRKG pathways as compared to control, except for *nNos* and *Gucy1a3*. T supplementation significantly increased the expression of all the genes that were related to NO signaling, except for *nNos* (Fig. 2, panels A-to-E). The concomitant addition of letrozole did not significantly alter T alone-induced effect. OVX also induced a down-regulation of *Pde5a* mRNA expression (Fig. 2, panel F), which was prevented by T (with or without letrozole). Moreover, OVX induced a significant reduction of mRNA expression of the smooth muscle-specific gene *Acta2* (*p* < 0.05 vs. intact female), that was completely normalized by T, either alone or in combination with letrozole (both *p* < 0.01 vs. OVX; Fig. 2, panel G). On the contrary, E2 administration did not affect OVX-induced reduction of all the aforementioned genes (Fig. 2).

### Relaxant effect of Ach on distal vagina strips from the different experimental groups

**Fig. 3:**
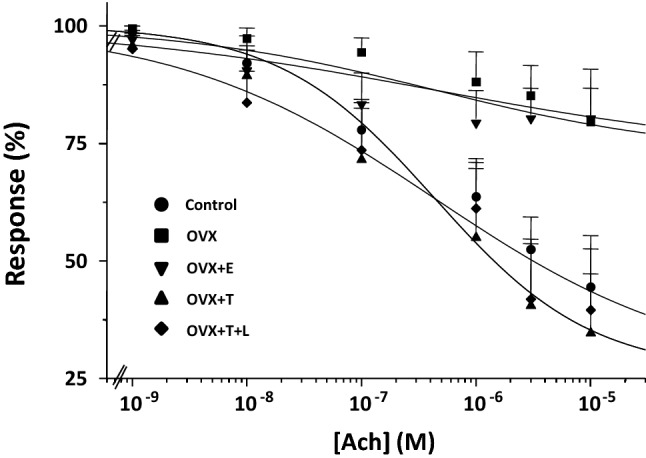
Smooth muscle relaxation elicited by increasing concentrations of Ach in vagina strips pre-contracted with NA from Control (*n* = 13), OVX (*n* = 11), OVX + E (*n* = 8), OVX + T (*n* = 7) and OVX + T + L (*n* = 6). Ordinate: Response to Ach is expressed as a percentage of contraction versus maximum contraction (set at 100%) obtained with 3 µM NA; abscissa: increasing concentrations of Ach (1 nM to 10 µM). Each point represents the mean ± s.e.m. of *n* samples for each group

**Fig. 4:**
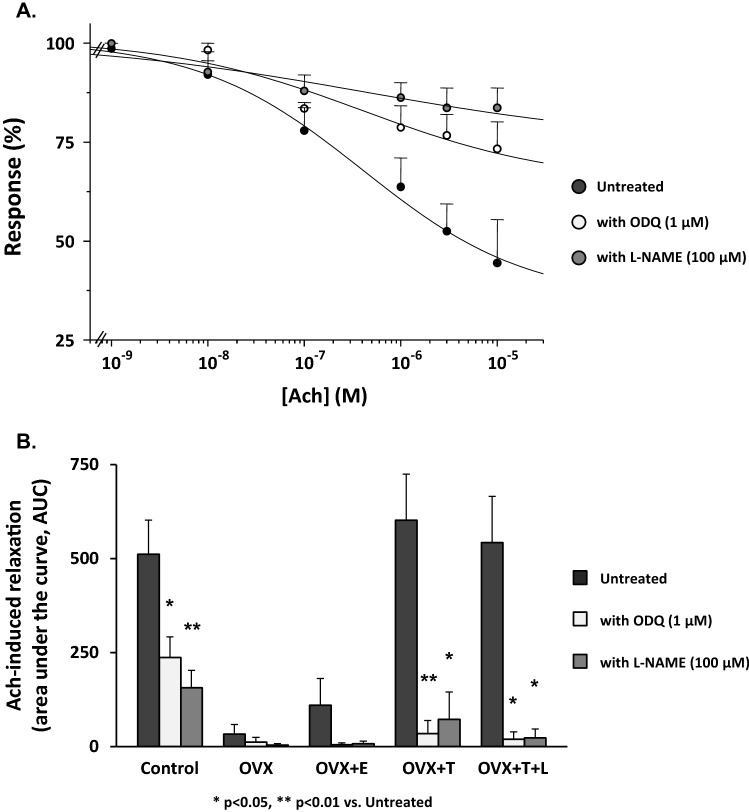
Panel **A** Relaxant effect induced by increasing concentrations of Ach (1 nM to 10 µM) in strips of vagina pre-contracted with 3 µM NA in Control samples left untreated (*n* = 13) or treated in vitro with either 1 µM ODQ (*n* = 4) or 100 µM L-NAME (*n* = 4). Response to Ach is expressed as a percentage of contraction versus maximum contraction (set at 100%) obtained with 3 µM NA. Panel **B** Area under the curve (AUC) of Ach-induced relaxation in each experimental group, left untreated or treated with either 1 µM ODQ or 100 µM L-NAME (at least 3 samples per group) *p < 0.05, **p < 0.01 vs. untreated

We next investigated the effect of the different hormonal conditions on the relaxant response to Ach by performing a series of dose–response curves in noradrenaline (NA)-precontracted vaginal strips (Fig. 3). Ach relaxed in a dose-dependent manner the vaginal strips obtained from the different experimental conditions with similar IC_50_s (shared IC_50_ = 0.44 ± 0.16 µM, *p* = 0.573). Maximal relaxation was obtained in control strips (*E*_max_ = 73.6 ± 15.7%), whereas ovariectomy significantly decreased *E*_max_ (*E*_max_ = 27.4 ± 1.6%, *p* < 0.0001 vs. control). In vivo estrogen administration to ovariectomized rats did not significantly restore Ach responsiveness up to the control levels, resulting in an *E*_max_ that was like that of ovariectomy alone (*p* = 0.86). In contrast, substitution with testosterone, with or without letrozole, to ovariectomized rats completely restored Ach responsiveness to a level not different from that observed in control strips (*p* = 0.79). We also tested the effect of in vitro incubation with 1 µM ODQ or with 100 µM L-NAME in the different experimental groups. Figure 4 (panel A) shows the results in control vaginal strips treated or not with the aforementioned compounds. Maximal relaxation was obtained in the untreated vaginal strips (*E*_max_ = 64.9 ± 16.9%), while either ODQ or L-NAME significantly smoothed Ach responsiveness (*E*_max_ = 35.2 ± 3.0% and *E*_max_ = 25.0 ± 1.4%, respectively, both *p* < 0.0001). Similar experiments were performed in vaginal samples obtained from the other experimental groups. Figure 4 (panel B) shows the area under the curve (AUC) of the relaxant response to Ach with or without in vitro exposure to ODQ or L-NAME in each experimental group.

### Relaxant effect of NO-donor, SNP, on distal vagina strips from the different experimental groups

**Fig. 5:**
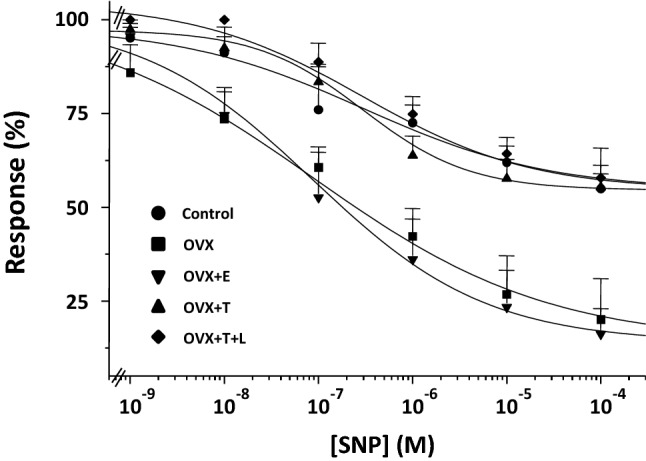
Smooth muscle relaxation elicited by increasing concentrations of SNP in distal vagina strips pre-contracted with NA from Control (*n* = 5), OVX (*n* = 5), OVX + E (*n* = 9), OVX + T (*n* = 7) and OVX + T + L (*n* = 5). Ordinate: Response to SNP is expressed as a percentage of contraction versus maximum contraction (set at 100%) obtained with 3 µM NA; abscissa: increasing concentrations of SNP (1 nM to 100 µM). Each point represents the mean ± s.e.m. of *n* samples (see above) for each group

**Fig. 6:**
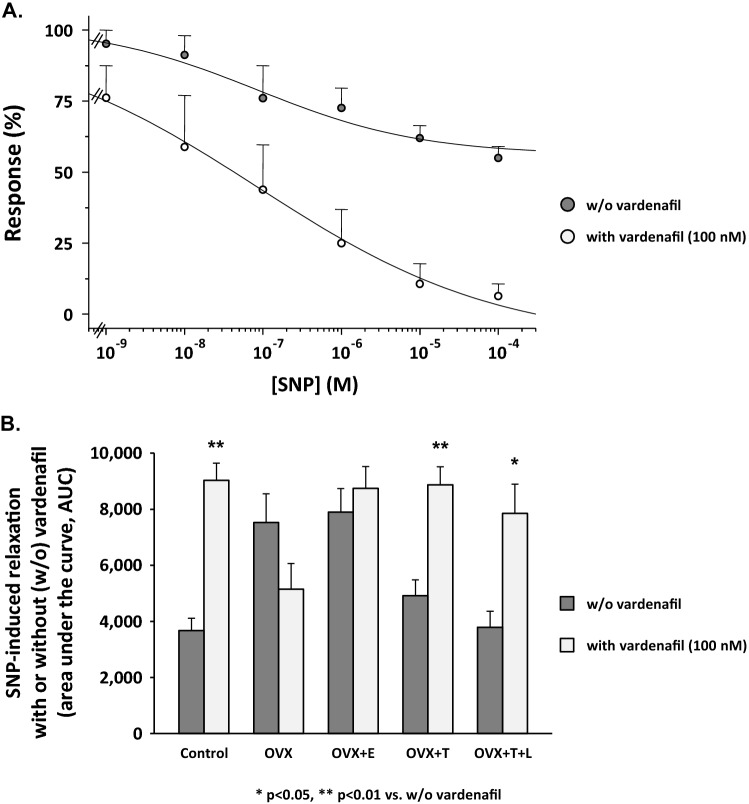
Panel **A** Relaxant effect induced by increasing concentrations of SNP (1 nM to 100 µM) in strips of vagina pre-contracted with 3 µM NA in Control samples with (*n* = 5) or without (w/o; *n* = 5) vardenafil (100 nM) in vitro. Response to SNP is expressed as a percentage of contraction versus maximum contraction (set at 100%) obtained with 3 µM NA. Panel **B** Area under the curve (AUC) of SNP-induced relaxation in each experimental group, with or without (w/o) vardenafil 100 nM (at least 3 samples per group). *p < 0.05, **p < 0.01 vs. w/o vardenafil

Figure 5 shows the effect of in vitro incubation with increasing concentrations of sodium nitroprusside (SNP) in NA-precontracted vagina from the different experimental groups. In control vaginal strips, SNP induced a maximal relaxation of 45.4 ± 1.9% with IC_50_ = 0.29 ± 0.10 µM. Results from vaginal strips from ovariectomized rats treated with testosterone, with or without letrozole, were not statistically different from those obtained in control rats. In contrast, in untreated ovariectomized rats, as well as in those treated with estradiol, maximal relaxation was almost the double (86.0 ± 19.7%), with a threefold lower IC_50_ = 0.09 ± 0.03 µM than in the first group of relaxation curves (see above, *p* = 0.001 and *p* = 0.017 vs. controls, respectively). Preincubation with vardenafil (100 nM) of vaginal strips from control rats induced a complete relaxation (100%, *p* < 0.0001 vs. without vardenafil), without changing IC_50_ = 0.08 ± 0.05 µM (Fig. 6, panel A). The area under the curve of SNP with or without vardenafil in the different experimental groups is shown in Fig. 6 (panel B). In control rats, vardenafil doubled the area under the curve of SNP. Similar results were obtained in ovariectomized rats treated with testosterone, but not in untreated ovariectomized rats or in those treated with estradiol, where the vardenafil-induced increase was not statistically significant.

## Discussion

We here demonstrated that T, independently from its conversion into estrogens, exerts relevant effects on vaginal tissue, directly modulating both endothelium-dependent and -independent relaxation. In particular, OVX exerted a powerful inhibitory effect on the Ach-induced relaxation of vaginal smooth muscle strips, which was normalized by in vivo treatment with T (either alone or in combination with letrozole). OVX also induced a hyper-responsiveness to SNP which was almost unaffected by co-administration of the PDE5 inhibitor, vardenafil. Interestingly, treatments with T, with or without letrozole, completely normalized responsiveness to SNP, while restoring the ability of vardenafil to potentiate SNP-induced relaxation. These positive effects of T, either on the endothelium-dependent (Ach) or -independent (SNP) relaxation, were not affected by co-administration with letrozole which completely abrogated T conversion into estrogens. Results from in vitro contractility studies were corroborated by the mRNA expression analysis of genes related to NO/cGMP/PRKG pathway. Finally, these findings further reinforce the concept that T, independently from its aromatization into E2, exerts a critical role for vaginal health by maintaining the integrity of the relaxant machinery in the vaginal muscular wall. In line with this view, 17βE_2_ treatment was not able to revert any of the OVX-induced effects.

Basic understanding of the molecular mechanisms of vagina function, as well as its regulation by sex steroids, is an area of great interest, because of its clinical implications, but still relatively overlooked. Earlier studies have clearly demonstrated that a decrease of ovarian steroids in natural and surgical menopausal women induced structural changes by shortening and narrowing the vagina [29, 30]. Interestingly, in our experimental animal model, the analysis by real-time RT-PCR shows a significant reduction, induced by OVX, of the mRNA expression of the functional smooth muscle marker α-SMA (*Acta2*). Accordingly, the Masson's trichrome staining performed in the distal vaginal sections derived from the OVX animal group, shows a large structural inhomogeneity of the muscularis mucosa, characterized by a smooth muscle component deranged and lax, combined with a clear reduction of the epithelial thickness, thus confirming a phenomenon already described in the literature [31].

However, most studies have focused on the effect of estradiol supplementation in modulating vaginal physiology [32, 33], but only a few have investigated the effect of T on vagina, and especially on its muscular compartment. In particular, T treatment in OVX rats positively regulated either electrical field stimulation (EFS)- or VIP-mediated relaxation in the distal vagina [20]. T supplementation in OVX animals upregulated nitric oxide synthase thus increasing vaginal blood flow after pelvic nerve stimulation [19, 34]. Moreover, T, but not E2, administration to OVX rats, normalized vaginal tissue weight, indicating that T more than estradiol plays an important role in exerting a trophic effect on the vagina muscular compartment [34]. In this sense, our data confirm these results, since the immunohistochemical analysis performed in the distal vagina samples show that treatments with T and T + L strongly counteract the tissue structural alterations induced by OVX, albeit without significant effects on the restoration of the vaginal epithelium. On the contrary, treatment with E2 completely restores the epithelial trophism, without significant effects on the smooth muscle construct. Confirming these data, in our study the mRNA expression of smooth muscle-related gene Acta2, significantly reduced by OVX, is strongly upregulated by T and T + L treatments but not by E2 administration. However, to date no studies on the effect of T on the most important pathways related to (Ach/NO-dependent) relaxant activities have been carried out.

Interestingly, we found that all the genes related to NO/cGMP/PRKG/PDE5 pathway were expressed in the distal vagina, with a profile that appears similar to that previously found in the clitoris in the same animal model [18]. Moreover, the entire NO/cGMP/PRKG/PDE5 pathway was dramatically modulated by T. Indeed, as previously observed in the clitoris [18], even in vagina ovariectomy significantly compromised relaxation induced by increasing concentration of Ach; this alteration was associated with a reduction of mRNA expression of key genes related to NO production (*eNos*) and signaling (*Gucy1a3*, *Gucy1b3*, and *Prkg1*). In line with these findings, we also observed that OVX significantly reduced the efficacy of NOS inhibitor (L-NAME) or of the guanylate cyclase inhibitor (ODQ) in disrupting Ach-induced relaxation. Treating OVX rats with T completely normalized not only all of the aforementioned NO-related genes but also impaired sensitivity to Ach along with the efficacy of the two NO-signaling inhibitors (L-NAME and ODQ). Therefore, it could be postulated that not only a reduced NO production but also an impaired activity of its downstream effectors were the main underlying mechanisms of the hypo-responsiveness to Ach observed in OVX rats, which were completely normalized by T treatment. Vaginal strips from OVX rats were also more sensitive to SNP-induced relaxation and less responsive to vardenafil, suggesting that sex steroids deprivation might down-regulate PDE5 activity. SNP, a NO donor, is used to assess the endothelium-independent relaxation while by passing NO formation through the direct activation of smooth muscle guanylate cyclase (GC) and the subsequent increase of cGMP. In OVX rats, vardenafil did not potentiate SNP induced relaxation, while its effect was evident in intact rats. Hypo-responsiveness to vardenafil is most probably due to a down-regulation of PDE5, the molecular target of PDE5 inhibitors, as we observed in OVX rats. Accordingly, T supplementation (with or without letrozole), but not 17β-estradiol, completely restored *Pde5a* expression, along with responsiveness to vardenafil, and normalized SNP-induced relaxation. Therefore, the present study also demonstrates that PDE5 is present and biologically active in the rat distal vagina while being positively modulated by androgen receptor (AR) activation. The presence of PDE5 in the vagina has been previously demonstrated by others in some animal species [35, 36], including the human one [15]. However, our data originally indicate that PDE5 is functional, and it might play a physiological role in controlling nitric oxide-cGMP-regulated vaginal smooth muscle relaxation. These data also corroborated the hypothesis that the PDE5 inhibitors might represent an interesting possibility for female sexual dysfunctions (FSD) [15, 37, 38]. Our results are also in line with some clinical report demonstrating a positive effect of PDE5 inhibitor treatment, when combined with testosterone, on FSD [39].

Interestingly, evidence that coadministration with letrozole did not modulate T effects either on endothelium-dependent and -independent relaxation as well as on genes related to NO-downstream signaling, indicates that aromatization is not necessary for ensuring androgenic activity in the smooth muscle compartment of distal vagina.

T is an essential hormone for women while being the obligatory precursor of estrogen by cytochrome P450 aromatase conversion. However, it has been already documented that co-administration of an aromatase inhibitor with T therapy in postmenopausal women did not affect the positive effect of T on arousal [4]. Similar results were obtained also in other animal studies. In fact, in OVX rats, T administration, even at supraphysiological concentrations, was not able either to change plasma estradiol levels or to normalize atrophic vaginal epithelium, when compared to vehicle infused OVX rats [34]. Therefore, taken together, these results exclude an effect dependent on the aromatization of androgen in the vagina. A direct effect of T was also exerted in the clitoris vascular bed, where co-administration of the aromatase inhibitor with T did not blunt T-induced positive effects on Ach-induced relaxation and on genes related to NO signaling [18]. Herein, we further reinforce the concept that androgens, more than estrogen, are key mediators in the vagina. Indeed, we recently demonstrated that aromatase expression and activity is almost absent in the human vagina while possessing all the enzymes for local T production and conversion into the androgen receptor superagonist, DHT [8]. This evidence substantiated the concept of intracrinology in the human vagina [8], similarly to what previously found in other animal species [40]. Androgens also possess anti-inflammatory properties in the human vagina. It has been reported that treatment with DHT significantly attenuated the inflammatory response induced by canonical inflammatory stimuli in isolated smooth muscle cells from the human vagina [9]. Overall, our data showing a strong effect of T on vaginal relaxation and related molecular machinery further highlights the extremely high androgen dependency of vaginal muscular physiology [41]. In contrast, we did not observe any significant effect of estradiol supplementation on vaginal smooth muscle relaxation induced by either Ach or SNP, and NO/cGMP relaxant machinery as compared to OVX.

However, there are some limitations to the study that should be recognized.

First, it would also be appropriate to add, in the study design, a subgroup of sham-operated control animals, for more correct feedback in the evaluation of the treatment effects in each experimental group. In addition, the experimental methods could be improved to reduce the number of animals employed. However, this animal model, albeit with its limitations, represents the most validated for the studies carried out and cannot, at the moment, be replaced by in vitro models.

It will also be important to finalize further studies to investigate the effects of sex steroids on contractility of vaginal smooth muscle, thus enabling to evaluate the contribution of this important physiological process to vaginal function.

Therefore, the often-overlooked effect of androgens in vagina appears of paramount importance to promote good organ functioning. This new concept might provide support for the concomitant use of androgens in the treatment of GSM and/or sexual arousal disorders related to ageing related-hormonal deficiency.

## Abbreviations

Ach: Acetylcholine
ANOVA: Analysis of variance
cDNA: Complementary DNA
DHT: Dihydrotestosterone
E2: 17β-estradiol
EFS: Electrical field stimulation
FSD: Female sexual dysfunction
sGC: Soluble guanylate cyclase
GDP: Guanosine-5′-diphosphate
cGMP: Cyclic guanosine monophosphate
GSM: Genitourinary syndrome of menopause
GTP: Guanosine-5′-triphosphate
L: Letrozole
L-NAME: Nω-Nitro-l-arginine methyl ester hydrochloride
NA: Noradrenaline
NO: Nitric oxide
eNOS: Endothelial nitric oxide synthase
OVX: Ovariectomy
PRKG: cGMP-dependent protein kinase
RT-PCR: Reverse transcriptase-polymerase chain reaction
SD: Sprague–Dawley
T: Testosterone
VIP: Vasoactive intestinal polypeptide

## References

[1] Davis SR, Wahlin-Jacobsen S (2015). Testosterone in women—the clinical significance. Lancet Diabetes Endocrinol.

[2] Simon JA, Goldstein I, Kim NN, Davis SR, Kellogg-Spadt S, Lowenstein L, Pinkerton JV, Stuenkel CA, Traish AM, Archer DF, Bachmann G, Goldstein AT, Nappi RE, Vignozzi L (2018). The role of androgens in the treatment of genitourinary syndrome of menopause (GSM): International Society for the Study of Women's Sexual Health (ISSWSH) expert consensus panel review. Menopause.

[3] Simon J, Braunstein G, Nachtigall L, Utian W, Katz M, Miller S, Waldbaum A, Bouchard C, Derzko C, Buch A, Rodenberg C, Lucas J, Davis S (2005). Testosterone patch increases sexual activity and desire in surgically menopausal women with hypoactive sexual desire disorder. J Clin Endocrinol Metab.

[4] Davis SR, Goldstat R, Papalia MA, Shah S, Kulkarni J, Donath S, Bell RJ (2006). Effects of aromatase inhibition on sexual function and well-being in postmenopausal women treated with testosterone: a randomized, placebo-controlled trial. Menopause.

[5] Panay N, Al-Azzawi F, Bouchard C, Davis SR, Eden J, Lodhi I, Rees M, Rodenberg CA, Rymer J, Schwenkhagen A, Sturdee DW (2010). Testosterone treatment of HSDD in naturally menopausal women: the ADORE study. Climacteric.

[6] Panjari M, Davis SR (2011). Vaginal DHEA to treat menopause related atrophy: a review of the evidence. Maturitas.

[7] Archer DF (2015). Dehydroepiandrosterone intra vaginal administration for the management of postmenopausal vulvovaginal atrophy. J Steroid Biochem Mol Biol.

[8] Cellai I, Di Stasi V, Comeglio P, Maseroli E, Todisco T, Corno C, Filippi S, Cipriani S, Sorbi F, Fambrini M, Petraglia F, Scavello I, Rastrelli G, Acciai G, Villanelli F, Danza G, Sarchielli E, Guarnieri G, Morelli A, Maggi M, Vignozzi L (2021). Insight on the intracrinology of menopause: androgen production within the human vagina. Endocrinology.

[9] Maseroli E, Cellai I, Filippi S, Comeglio P, Cipriani S, Rastrelli G, Rosi M, Sorbi F, Fambrini M, Petraglia F, Amoriello R, Ballerini C, Lombardelli L, Piccinni MP, Sarchielli E, Guarnieri G, Morelli A, Maggi M, Vignozzi L (2020). Anti-inflammatory effects of androgens in the human vagina. J Mol Endocrinol.

[10] Burnett AL, Calvin DC, Silver RI, Peppas DS, Docimo SG (1997). Immunohistochemical description of nitric oxide synthase isoforms in human clitoris. J Urol.

[11] Cellek S, Moncada S (1998). Nitrergic neurotransmission mediates the non-adrenergic non-cholinergic responses in the clitoral corpus cavernosum of the rabbit. Br J Pharmacol.

[12] Vemulapalli S, Kurowski S (2000). Sildenafil relaxes rabbit clitoral corpus cavernosum. Life Sci.

[13] Park JK, Kim JU, Lee SO, Hwang PH, Yi HK, Kim YG, Cho KW (2002). Nitric oxide cyclic GMP signaling pathway in the regulation of rabbit clitoral cavernosum tone. Exp Biol Med (Maywood).

[14] Park K, Moreland RB, Goldstein I, Atala A, Traish A (1998). Sildenafil inhibits phosphodiesterase type 5 in human clitoral corpus cavernosum smooth muscle. Biochem Biophys Res Commun.

[15] D'Amati G, di Gioia CR, Bologna M, Giordano D, Giorgi M, Dolci S, Jannini EA (2002). Type 5 phosphodiesterase expression in the human vagina. Urology.

[16] Ückert S, Ellinghaus P, Albrecht K, Jonas U, Oelke M (2007). Expression of messenger ribonucleic acid encoding for phosphodiesterase isoenzymes in human female genital tissues. J Sex Med.

[17] Ückert S, Oelke M, Albrecht K, Breitmeier D, Kuczyk MA, Hedlund P (2011). Expression and distribution of key enzymes of the cyclic GMP signaling in the human clitoris: relation to phosphodiesterase type 5 (PDE5). Int J Impot Res.

[18] Comeglio P, Cellai I, Filippi S, Corno C, Corcetto F, Morelli A, Maneschi E, Maseroli E, Mannucci E, Fambrini M, Maggi M, Vignozzi L (2016). Differential effects of testosterone and estradiol on clitoral function: an experimental study in rats. J Sex Med.

[19] Traish AM, Kim NN, Huang YH, Min K, Munarriz R, Goldstein I (2003). Sex steroid hormones differentially regulate nitric oxide synthase and arginase activities in the proximal and distal rabbit vagina. Int J Impot Res.

[20] Kim NN, Min K, Pessina MA, Munarriz R, Goldstein I, Traish AM (2004). Effects of ovariectomy and steroid hormones on vaginal smooth muscle contractility. Int J Impot Res.

[21] Idris AI (2012). Ovariectomy/orchidectomy in rodents. Methods Mol Biol.

[22] Liang CC, Lee TH, Chang SD (2013). Effects of sex hormones on cell proliferation and apoptosis in the urinary bladder muscle of ovariectomized rat. Taiwan J Obstet Gynecol.

[23] Dehghan F, Muniandy S, Yusof A, Salleh N (2014). Sex-steroid regulation of relaxin receptor isoforms (RXFP1 & RXFP2) expression in the patellar tendon and lateral collateral ligament of female WKY rats. Int J Med Sci.

[24] Song X, Zhao P, Wang G, Zhao X (2014). The effects of estrogen and androgen on tear secretion and matrix metalloproteinase-2 expression in lacrimal glands of ovariectomized rats. Invest Ophthalmol Vis Sci.

[25] Percie du Sert N, Hurst V, Ahluwalia A, Alam S, Avey MT, Baker M, Browne WJ, Clark A, Cuthill IC, Dirnagl U, Emerson M, Garner P, Holgate ST, Howells DW, Karp NA, Lazic SE, Lidster K, MacCallum CJ, Macleod M, Pearl EJ, Petersen O, Rawle F, Peynolds P, Rooney K, Sena ES, Silberberg SD, Steckler T, Wurbel H (2020). The ARRIVE guidelines 2.0: updated guidelines for reporting animal research. PLoS Biol.

[26] Giraldi A, Alm P, Werkstrom V, Myllymaki L, Wagner G, Andersson KE (2002). Morphological and functional characterization of a rat vaginal smooth muscle sphrincter. Int J Impot Res.

[27] Livak KJ, Schmittgen TD (2001). Analysis of relative gene expression data using real-time quantitative PCR and the 2(-Delta C(T)) Method. Methods.

[28] DeLean A, Munson PJ, Rodbard D (1978). Simultaneous analysis of families of sigmoidal curves: application to bioassay, radioligand assay, and physiological dose-response curves. Am J Physiol.

[29] Forsberg JG (1995). A morphologist's approach to the vagina–age-related changes and estrogen sensitivity. Maturitas.

[30] Park K, Ahn K, Lee S, Ryu S, Park Y, Azadzoi KM (2001). Decreased circulating levels of estrogen alter vaginal and clitoral blood flow and structure in the rabbit. Int J Impot Res.

[31] Onol FF, Ercan F, Tarcan T (2006). The effect of ovariectomy on rat vaginal tissue contractility and histomorphology. J Sex Med.

[32] Ballagh SA (2005). Vaginal hormone therapy for urogenital and menopausal symptoms. Semin Reprod Med.

[33] Nappi RE, Lachowsky M (2009). Menopause and sexuality: prevalence of symptoms and impact on quality of life. Maturitas.

[34] Traish AM, Kim SW, Stankovic M, Goldstein I, Kim NN (2007). Testosterone increases blood flow and expression of androgen and estrogen receptors in the rat vagina. J Sex Med.

[35] Pace G, Palumbo P, Miconi G, Silvestri V, Cifone MG, Vicentini C (2011). PDE-5 and NOS II mRNA expression in menopausal women: a molecular biology study. World J Urol.

[36] Cho KJ, Lee KS, Choo MS, Seo JT, Kim JH, Choi JB, Oh SJ, Kim JC (2017). Expressions of vaginal endothelial nitric oxide synthase and phosphodiesterase 5 in female sexual dysfunction: a pilot study. Int Urogynecol J.

[37] Chivers ML, Rosen RC (2010). Phosphodiesterase type 5 inhibitors and female sexual response: faulty protocols or paradigms?. J Sex Med.

[38] Poels S, Bloemers J, van Rooij K, Goldstein I, Gerritsen J, van Ham D, van Mameren F, Chivers M, Everaerd W, Koppeschaar H, Olivier B, Tuiten A (2013). Toward personalized sexual medicine (part 2): testosterone combined with a PDE5 inhibitor increases sexual satisfaction in women with HSDD and FSAD, and a low sensitive system for sexual cues. J Sex Med.

[39] Bloemers J, Gerritsen J, van Rooij K, de Leede L, van der Geest R, Frijlink HW, Koppeschaar HPF, Olivier B, Tuiten A (2019). The effect of food on the pharmacokinetics of sildenafil after single administration of a sublingual testosterone and oral sildenafil combination tablet in healthy female subjects. J Sex Med.

[40] Bertin J, Dury AY, Ouellet J, Pelletier G, Labrie F (2014). Localization of the androgen-synthesizing enzymes, androgen receptor, and sex steroids in the vagina: possible implications for the treatment of postmenopausal sexual dysfunction. J Sex Med.

[41] Maseroli E, Vignozzi L (2020). Testosterone and vaginal function. Sex Med Rev.

